# Treatment of refractory Yao syndrome with canakinumab

**DOI:** 10.1016/j.jdcr.2022.08.035

**Published:** 2022-08-28

**Authors:** Caroline J. Brailsford, Fatima Khamdan, Dirk M. Elston

**Affiliations:** a College of Medicine, Medical University of South Carolina, Charleston, South Carolina; b Department of Dermatology and Dermatologic Surgery, Medical University of South Carolina, Charleston, South Carolina

**Keywords:** NOD2, NOD2 mutation, NOD2-associated autoinflammatory disease, nucleotide-binding oligomerization domain 2, Yao syndrome, CD, Crohn’s disease, BS, Blau Syndrome, IL, interleukin, NOD2, nucleotide-binding oligomerization domain 2, YAOS, Yao syndrome

## Introduction

Yao Syndrome (YAOS), formerly designated nucleotide-binding oligomerization domain 2 (NOD2)-associated autoinflammatory disease, consists of recurrent fever, rash, arthritis, distal extremity swelling, gastrointestinal symptoms, and sicca-like symptoms (YAOS; OMIM 617321).^1^^,^^2^ YAOS is a genetically complex disorder involving multiple loci of the NOD2 gene, which encodes a pattern recognition receptor important in the innate immune response.^3^ NOD2 gene variants have also been implicated in Blau Syndrome (BS) and Crohn’s Disease (CD).^2^ Therefore, diagnosis relies on genetics in conjunction with clinical presentation and exclusion of other systemic autoinflammatory diseases.^4^ Although YAOS can affect both the pediatric and adult populations, the onset of the disease is most common between 20 and 50 years with a female-to-male ratio of 2:1.^2^ Autoinflammatory diseases may respond to oral glucocorticoids, dapsone, colchicine, and sulfasalazine. Biologic therapy with interleukin (IL)-1, IL-6, and tumor necrosis factor-alfa inhibitors is useful in some patients with refractory disease.^2^^,^^5^^,^^6^ We report a 27-year-old male patient with YAOS refractory to prednisone, colchicine, dapsone, and anakinra with suppression of disease following treatment with canakinumab IL-1β inhibitor therapy.

## Case report

**Fig 1:**
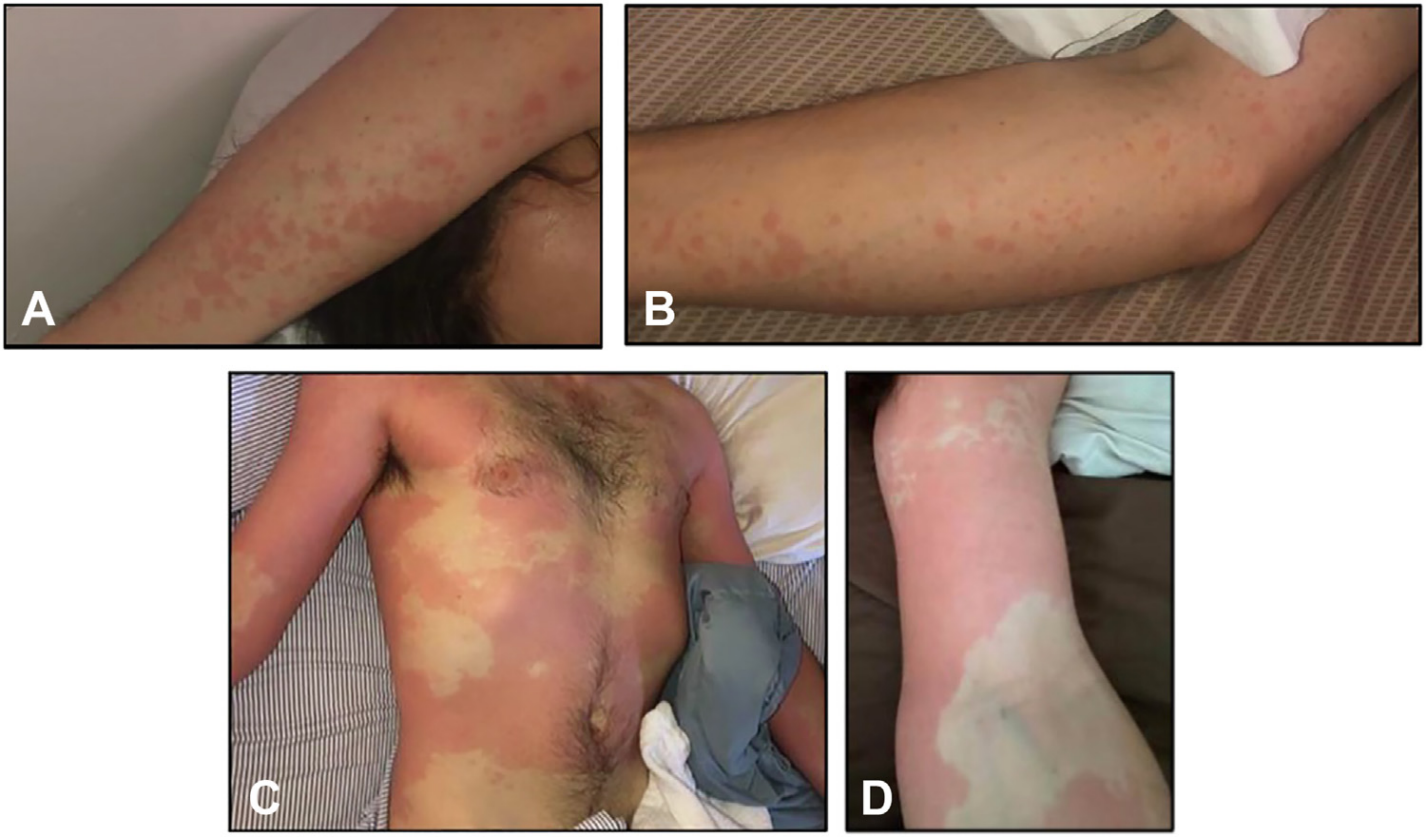
**A-D**. A 27-year-old Caucasian man presented to the dermatology clinic with a history of episodic fever and recurrent tender erythematous rash with significant body involvement.

A 27-year-old White man presented to the dermatology clinic with a history of episodic fever and recurrent tender erythematous rash with significant body involvement, especially of the trunk (Fig 1, *A*-*D*). The rash was reportedly triggered by psychologic stress and preceded by prodromal flu-like symptoms, low-grade fever, sore throat, and arthralgias. During these episodes, he experienced intermittent diarrhea and severe headache. Over time, flares worsened in severity, causing the patient to be bedbound during episodes. Symptoms persisted for 3 to 7 days before spontaneously resolving and recurred every 3 weeks.

**Fig 2:**
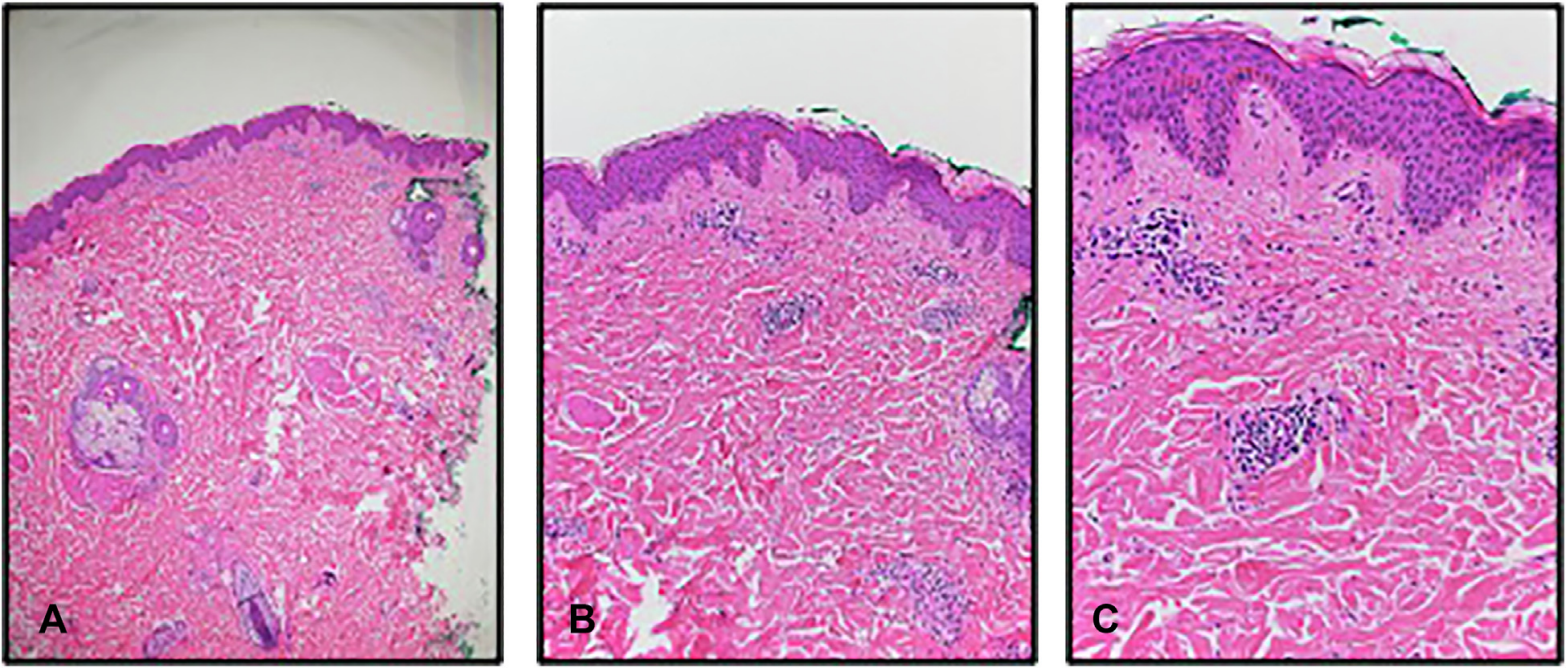
**A-C**. Biopsies were taken from a patient with Yao Syndrome showing sparse superficial polymorphic inflammatory infiltrate consisting mainly of lymphocytes; few eosinophils and neutrophils. No evidence of vasculitis. (**A,B,** and **C,** Hematoxylin-eosin stain; original magnifications: **A,** ×5; **B,** ×100 and **C,** ×20.)

Past medical history included seasonal allergic rhinitis with no family history of autoimmune disease. Skin biopsies performed by his previous dermatologist demonstrated perivascular neutrophilic infiltrate with no evidence of leukocytoclastic vasculitis (Fig 2, *A*-C). The patient trialed multiple antihistamines, prednisone, and more recently, monthly omalizumab 125-mg/mL injections with no relief. Further laboratory workup was notable for mildly decreased C4 (12.8 mg/mL; reference range 15-57) and negative for antinuclear antibodies, antineutrophil cytoplasmic antibodies, rheumatoid factor, and other complement disturbances. Thyroid hormones, C-reactive protein, and erythrocyte sedimentation rate were within normal limits. Antibodies to deamidated gliadin and transglutaminase were negative and serum levels, IgA, serotonin, chromogranin A, and tryptase were within normal limits. Screenings for hepatitis, HIV, toxocara, and tuberculosis were negative. Urinary catecholamines, metanephrines, and 5-hydroxyindoleacetic acid were within normal limits. The chronic urticaria index was within normal limits. The patient was on omalizumab at the time laboratory studies were performed.

A gene assay for autoinflammatory syndromes was performed, revealing heterozygous c.3019dup (p.Leu1007Profs∗2) found on exon 11 of the NOD2 gene, consistent with YAOS. A colonoscopy was performed with no evidence of inflammatory bowel disease. The patient was then initiated on trials of colchicine and sulfasalazine with only minor improvement. He was switched to daily anakinra 100 mg/0.67 mL injections but continued to suffer recalcitrant skin flares and debilitating arthritis. Anakinra was switched to IL-1β inhibitor canakinumab 150 mg/mL monthly injections. The patient reports both decreased frequency and severity of episodes and is satisfied with the results.

## Discussion

YAOS often presents with recurrent and remitting flu-like symptoms followed by fever and erythematous plaques or patches.^4^ Our patient experienced symptoms that are commonly reported with YAOS: gastrointestinal manifestations (bloating, abdominal pain, and diarrhea), arthritis and arthralgias, lymphadenopathy, pharyngitis, headache, weight loss, and chronic pain causing physical impairment.^2^^,^^4^^,^^7^^,^^8^

Other reported features of YAOS include sicca-like symptoms, most commonly periorbital swelling, lower extremity swelling, oral ulcers, elevated erythrocyte sedimentation rate/C-reactive protein, leukocytosis, pericarditis, and pleuritis.^2^^,^^4^^,^^7^^,^^8^ Cerebral infarction has also been reported in the context of a YAOS-associated fever attack.^9^ Eyelid swelling is especially common, seen in >50% of patients with YAOS. Its absence in our case highlights disease variability and emphasizes recognizing its full spectrum of manifestations. Although 90% of Caucasians experience dermatitis, no Chinese patients with dermatitis have been reported, suggesting possible human leuokocyte antigen-mediated differences in disease expression.^8^

A broad differential should include other systemic autoinflammatory diseases, a group of disorders caused by chronic activation of the innate immune system. In contrast with classic autoimmune disorders, autoinflammatory conditions lack identifiable autoantibodies but rather comprise periodic fever syndromes with evanescent urticarial papules, plaques, figurate erythema, erysipelas-like eruptions, and sterile pustules, typically in association with. fever Elevated acute phase reactants, rash, serositis, arthritis, and lymphadenopathy are present in both autoinflammatory and autoimmune conditions.^10^ Yao et al^2^ developed a 6-gene panel to screen for periodic fever syndromes that may mimic YAOS (Table I). Typically, recurrent patchy erythema, distal extremity swelling, and eyelid swelling are more suggestive of YS than other hereditary periodic fever syndromes.^2^

**Table I:** Periodic fever syndrome gene panel^2^

Gene implicated	Associated periodic fever syndrome
MEFV	Familial Mediterranean fever
TNFRSF1A	TNF-receptor–associated periodic syndrome
NLRP3	Cryopyrin-associated periodic syndromes
MVK	Hyper-IgD syndrome (HIDS)/mevalonate kinase deficiency
NLRP12	NLRP12 autoinflammatory disease
NOD2	Yao Syndrome, Blau Syndrome, Crohn’s Disease

*TNF*, tumor necrosis factor; *IgD*, immunoglobulin D.

The NOD2 protein is a cytosolic receptor that serves in innate immune recognition of peptidoglycan or muramyl dipeptide components of bacterial cell walls and initiates an inflammatory response.^2^ The NOD2 gene, found on chromosome 16, is implicated in 3 periodic fever syndromes: BS, CD, and YAOS.

BS is linked to gain-of-function variants in the receptor’s central NOD domain on exon 4.^2^ Clinically, BS is primarily a pediatric disease characterized by a triad of granulomatous dermatitis, uveitis, and inflammatory arthritis.^8^ Development of disease in adults and the presence of fever, abdominal pain, and diarrhea are rarely seen.^2^ Moreover, panuveitis and choroiditis are frequently seen in BS but are uncommon in YAOS.^2^

CD has been associated with NOD2 loss of function mutations 1007fs, G908R, and R702W.^4^ These NOD2 variants, although present in <50% of patients with CD, overlap with variants identified in YAOS. The absence of bloody diarrhea may be more indicative of YAOS. However, an endoscopy examination is warranted to exclude CD if these gene variants are identified.^2^

Mutations IVS8+158 and R702W are the most frequently reported in YAOS, but other NOD2 variants of unknown significance have been identified, as seen in our patient.^4^ These are nearly all heterozygous and sporadically occurring, with 10% reporting positive family history.^2^ Interplay between genetics and environmental triggers is postulated to play a role in disease expression.^8^

Treatment in patients with mild disease may be achieved with short courses of oral glucocorticoids and daily sulfasalazine.^4^ In severe and refractory disease or patients with adverse reactions to first-line therapies, biologic therapy with IL-1 and IL-6 has been effective in controlling attacks.^2^^,^^4^ Colchicine has proved less effective in most patients although it is helpful in other autoinflammatory syndromes.^4^ Canakinumab and anakinra both function in the inhibition of the IL-1 pro-inflammatory cascade. Anakinra is an IL-1 receptor antagonist, competitively inhibiting the interaction with IL-1α and IL-1β and blocking downstream effects. Canakinumab is a fully humanized IgG1 monoclonal antibody to IL-1β which has been identified as an effective treatment option for inflammatory conditions in which anakinra has failed.^11^^,^^12^ We postulate that its longer duration of action plays a key role in its therapeutic benefit. Canakinumab has recently been identified as a treatment option in YAOS, administered subcutaneously in 150-mg injections every 4 to 8 weeks.^2^^,^^6^ Our patient presented with severe YAOS and responded to canakinumab after failure with multiple other agents.

## Conflicts of interest

None disclosed.
